# Whole genome analysis reveals the distribution and diversity of plasmid reservoirs of NDM and MCR in commercial chicken farms in China

**DOI:** 10.1128/spectrum.02900-24

**Published:** 2025-06-09

**Authors:** Xuan Wu, ZhiRong Zhang, Rong Xiang

**Affiliations:** 1 Key Laboratory of Environmental Chemistry and Ecotoxicology of Organic Pollutants of Chongqing, Chongqing Ecological and Environment Monitoring Center, Chongqing, China; 2 Precision Medicine Center, The Second Affiliated Hospital of Chongqing Medical University 585250, Chongqing, Chongqing, China; University of Pittsburgh School of Medicine, Pittsburgh, Pennsylvania, USA

**Keywords:** NDM, MCR, multidrug-resistant, chicken farms

## Abstract

The increase in multidrug-resistant (MDR) Enterobacteriaceae presents a significant challenge to clinical treatment, particularly in infections where carbapenems and colistin serve as the last-resort antimicrobial agents. In this study, we isolated 119 non-repetitive gram-negative bacteria from MacConkey medium supplemented with imipenem and colistin. The isolates were dominated by *Klebsiella pneumoniae* (58.0%, *n* = 69) and *Escherichia coli* (31.1%, *n* = 37). The predominant sequence types (STs) of *E. coli* were ST226, ST1286, and ST11738, whereas *K. pneumoniae* displayed ST152, ST395, and ST709 as major types. Genomic analysis identified *mcr-1/3/8/9* in 44 strains and *bla*_NDM_ in 63 strains across various species. IncX3 (*n* = 57) and IncFII (*n* = 5) were the most common *bla*_NDM-5_-carrying plasmid types. Several plasmid replicons were associated with *mcr* genes, including IncI2, IncX4, and novel plasmids. Remarkably, we discovered four combinations of *bla*_NDM_ and mcr co-occurrence in 28 isolates, including *bla*_NDM-5_/*mcr-1*, *bla*_NDM-5_/*mcr-3*, *bla*_NDM-5_/*mcr-8,* and *bla*_NDM-5_/*mcr-9*. Our findings reveal that chicken farms are significant reservoirs for both *bla*_NDM_ and *mcr* genes, with frequent co-occurrence of these resistance determinants. The presence of these genes alongside other resistance factors, such as *blaESBL*, highlights a critical public health risk. This study underscores the need for enhanced surveillance and intervention strategies to mitigate the spread of MDR pathogens from agricultural environments to clinical settings.

**IMPORTANCE:**

This study reveals that commercial poultry farms in China serve as critical reservoirs for MDR gram-negative bacteria harboring carbapenemase (*bla*_NDM_) and mobilized colistin resistance (mcr) genes. By analyzing 119 isolates, we uncovered extensive genetic diversity and plasmid-mediated co-occurrence of these resistance determinants, enabling bacteria to evade nearly all available treatments. Alarmingly, the horizontal transfer of resistance genes via highly mobile plasmids facilitates their spread across microbial communities and potentially into clinical settings. These findings underscore the urgent need to address antibiotic overuse in agriculture and strengthen surveillance under the One Health framework. The persistence of MDR pathogens in poultry environments highlights a significant risk for zoonotic transmission, emphasizing the necessity of coordinated interventions to curb the global antimicrobial resistance crisis.

## INTRODUCTION

Antimicrobial resistance (AMR) is now recognized as a critical global health threat, driven by the rapid increase in multidrug- and pandrug-resistant bacteria, particularly those resistant to last-resort antibiotics like carbapenems and colistin (1, 2). Among these, New Delhi metallo-β-lactamase-1 (NDM-1) is notable for its broad-spectrum resistance to almost all β-lactam antibiotics, including carbapenems. Since its discovery, 58 variants of NDM enzymes have been identified, with *bla*_NDM-1_ and *bla*_NDM-5_ remaining the most prevalent (3). In parallel, colistin resistance, mediated by plasmid-encoded *mcr* genes (*mcr-1* to *mcr-10*), has emerged globally, threatening the effectiveness of this last-line defense against carbapenem-resistant pathogens (2, 4, 5). The co-existence of carbapenem resistance (CR) and colistin resistance (col-R) in bacterial pathogens poses a serious public health threat. Although AMR has been extensively investigated in clinical settings (6, 7), agricultural environments—particularly poultry farms—are increasingly recognized as significant reservoirs of AMR (8). Bacterial strains harboring ARGs within these farms not only compromise food safety but also have the potential to disseminate into surrounding environments via aerosols and dust particles (9, 10). Moreover, the application of poultry manure as fertilizer may further facilitate the environmental spread of ARGs (11). Notably, there are a few reports on the prevalence of CR and col-R in poultry farms. This gap hinders our understanding of how these settings contribute to the global AMR crisis. In China, one of the world’s largest poultry producers, the use of antibiotics in farming practices has raised concerns about the role of poultry farms in harboring and disseminating AMR. Although several clinically important antibiotics, including colistin, have been banned in agricultural production in China, resistant bacteria are still being isolated from agricultural environments (12, 13). This suggests that although these antibiotics are banned, their historical use in agricultural production has induced or enriched large populations of resistant microorganisms in the agricultural environment, leading to irreversible impacts on microbial communities. The spread of these resistant bacteria and the transfer of their resistance genes within microbial communities will continue to threaten human health and complicate clinical infection treatments.

In this study, we focused on six unrelated commercial chicken farms in China to investigate the occurrence of CR and col-R gram-negative bacteria as part of ongoing resistance surveillance. A total of 119 representative CR/col-R isolates were collected, primarily from the farm environment, and analyzed through whole-genome sequencing (WGS). The sequencing revealed the phylogenetic diversity, plasmid types, multilocus sequence typing (MLST) profiles, and resistance gene content of the isolates. Key findings included the identification of multiple variants of *bla*_NDM_ and *mcr* genes, as well as the genomic contexts in which these genes were found. Notably, the co-occurrence of *bla*_NDM_ and *mcr* genes was observed, further highlighting the risk posed by poultry farms as reservoirs for these critical resistance determinants.

The results of this study demonstrate that commercial poultry farms in China are significant reservoirs of carbapenem- and colistin-resistant bacteria. The widespread presence of *bla*_NDM_ and *mcr* genes, often co-located on plasmids, underscores the urgent need for enhanced surveillance and monitoring of AMR in agricultural settings. These findings highlight the interconnection between human, animal, and environmental health, which is an essential component of the “One Health” framework, and emphasize the importance of addressing antibiotic overuse in farming practices to prevent the dissemination of AMR into the broader environment (14). This study provides valuable insights into the role of poultry farms in the global AMR crisis and reinforces the need for proactive measures to safeguard public health.

## MATERIALS AND METHODS

### Sampling and bacterial isolates

To ensure a representative sample, a total of 1,862 cloacal samples were randomly collected from six independent commercial chicken farms in China as part of a routine study on antimicrobial-resistant gram-negative bacteria. All the samples were sent to the laboratory within 24 h and preserved in a refrigerator at 4°C. Each isolate was cultured in 10 mL of brain heart infusion (BHI) broth at 37°C, and shaken at 180 r/min for 16 h. The cultures were inoculated onto MacConkey medium supplemented with 2 µg/mL imipenem and 2 µg/mL colistin to selectively isolate carbapenem and colistin-resistant strains, as these concentrations effectively suppress susceptible bacteria while allowing resistant strains to grow. From each sample plate, colonies displaying distinct morphologies were selected by assessing differences in size, shape, margin, elevation, texture, opacity, and color/lactose fermentation on MacConkey agar. These selected isolates underwent initial identification via 16S rRNA sequencing. Subsequently, taxonomic classification was confirmed using the GTDB-Tk (v0.3.0) software toolkit with default parameters, based on the Genome Taxonomy Database (15).

### Antimicrobial susceptibility testing

Antimicrobial susceptibility testing (AST) was performed for all isolates using the standard broth microdilution method in Mueller-Hinton Broth (MHB). Minimum inhibitory concentrations (MICs) were determined for a panel of antibiotics including imipenem, meropenem, colistin, cefotaxime, ceftazidime, chloramphenicol, gentamicin, tetracycline, trimethoprim, and amikacin. Interpretation of MICs was performed using the following criteria: for colistin, the European Committee on Antimicrobial Susceptibility Testing (EUCAST) guidelines (v12.0, 2022) were applied (susceptible ≤2 µg/mL; resistant >2 µg/mL); for all other antimicrobial agents tested, the Clinical and Laboratory Standards Institute (CLSI) guidelines (M100, 32nd Edition, 2022) and relevant species-specific breakpoints were used. Detailed susceptibility results are provided in Table S5.

### Whole-genome sequencing and assembly

Genomic DNA from all 119 strains was extracted using the QIAGEN Genome Kit (QIAGEN, Hilden, Germany) and sequenced using paired-end sequencing on the Illumina MiSeq platform (Illumina, San Diego, CA), following the manufacturer’s instructions. For each isolate, a minimum of 100× coverage of raw reads was achieved through Illumina sequencing. Trimmed sequences from the Illumina MiSeq were *de novo* assembled using SPAdes v3.0 (https://github.com/ablab/spades). Some of the contigs were very short. To improve the genetic context around the *mcr* and *bla*_NDM_ genes, we conducted a hybrid assembly on six strains using Oxford Nanopore MinION long reads for scaffolding and Illumina HiSeq for error correction. Genomic contigs were *de novo* assembled using the default settings in Canu (version 1.8) (16), and the assemblies were error-corrected with Pilon (version 1.22) (17).

### Bioinformatics analysis

Gene prediction and annotation were carried out using the prokaryote annotation tool Prokka combined with the BLAST program (http://blast.ncbi.nlm.nih.gov/Blast.cgi). Acquired antimicrobial resistance genes were identified in the whole-genome assemblies using ABRicate (v1.0.1, https://github.com/tseemann/abricate). Abricate was run using the Resfinder database (18) with the following thresholds: a minimum DNA identity of 70% and a minimum DNA coverage of 80%. Annotation of mobile elements was carried out using ISfinder (https://www-is.biotoul.fr/). Plasmid replicon types were determined by the PlasmidFinder tool at https://cge.food.dtu.dk/services/PlasmidFinder with default parameters (≥95% identity and ≥60% coverage). The plasmid and chromosomal locations of ARGs were predicted using PlasFlow (19). The multiple locus sequence typing (MLST) was performed using MLST (https://github.com/tseemann/mlst). PHASTER was used to predict the prophage population in each genome (http://phaster.ca/) employing its default parameters for identifying phage-related genes and scoring region completeness (20).

Comparison of genetic context in the different *mcr*-carrying and *bla*_NDM_-carrying contigs was performed with Easyfig (21). Contigs with similar genetic contexts were grouped together based on BLAST analysis against the National Center for Biotechnology Information (NCBI) databases.

### Phylogenetic analyses

Phylogenetic analysis of all the genomes was performed by the kSNP3 program (22) using the default parameter with the maximum-likelihood tree options. The Kchooser script of the kSNP3 program was used to determine the optimal value of k-mer size. Finally, the phylogenetic tree was accomplished using PhyML and visualized using the online web server Interactive Tree of Life platform (itol.embl.de) (23).

## RESULTS

### Widespread multidrug resistance gram-negative bacteria in Chinese poultry farms

**Fig 1:**
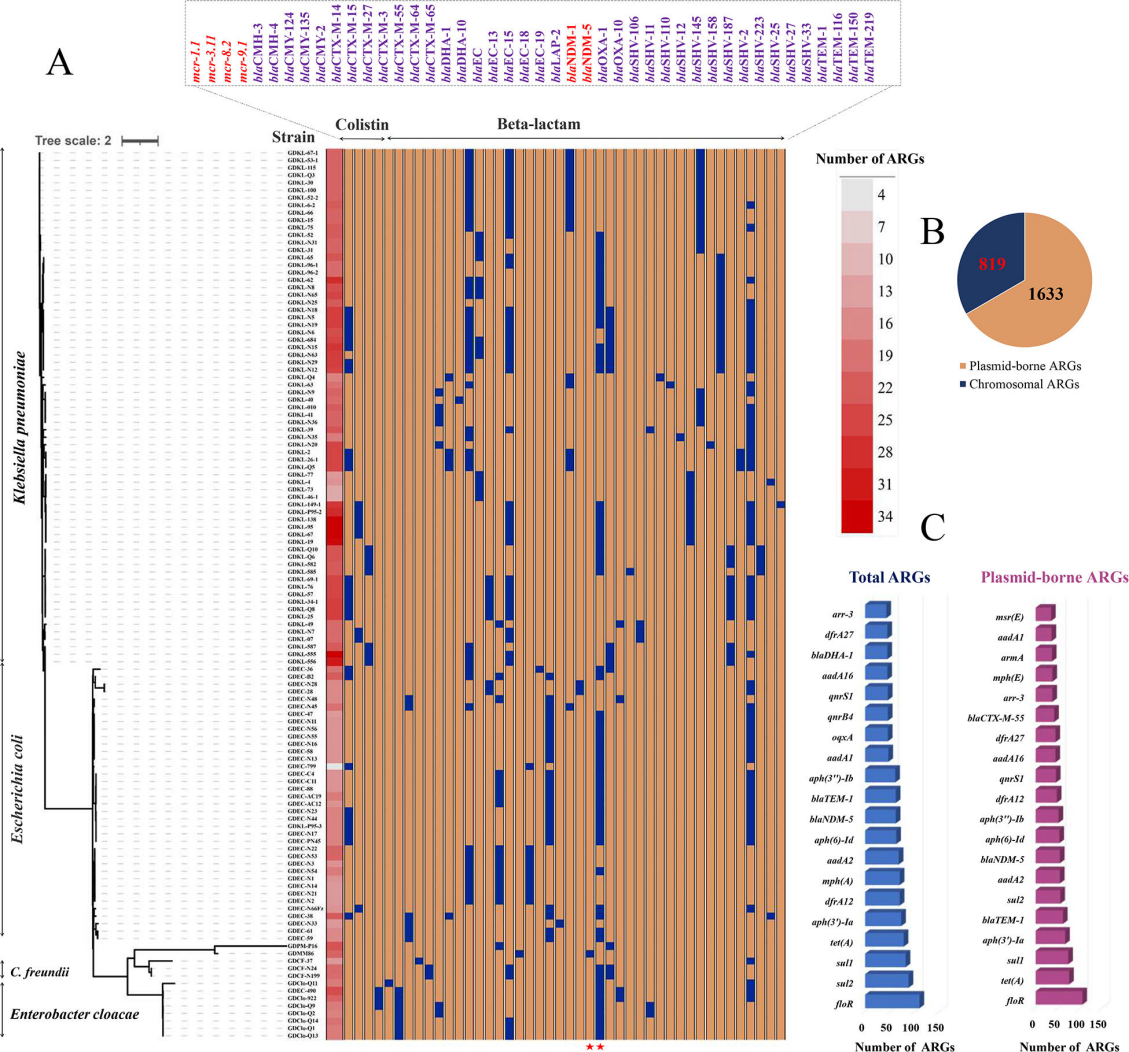
Phylogeny and basic information for the 119 gram-negative strains isolated from commercial chicken farms. (**A**) The phylogenetic relationship of 119 representative CR/col-R isolates, and their antibiotic resistance genes. The left phylogenetic tree was generated using the SNP-matrix algorithm provided by the kSNP3 tool. The presence of antibiotic resistance genes is highlighted in blue. (**B**) Number of putative plasmid-borne ARGs and chromosomal ARGs. (**C**) Distribution of the top 20 most prevalent ARGs-type and plasmid-borne ARGs in all 119 isolates.

We collected a total of 119 non-repetitive gram-negative isolates from commercial chicken farms between 2020 and 2022. Subsequent analysis revealed widespread multidrug resistance (MDR) among these isolates(Fig.1). The species identified and the number of isolates from each was as follows: *K. pneumoniae* (*n* = 69), *E. coli* (*n* = 37), *Enterobacter cloacae* (*n* = 8), *Citrobacter freundii* (*n* = 3), *Morganella morganii* (*n* = 1), and *Proteus mirabilis* (*n* = 1). Antimicrobial susceptibility testing revealed that 53% (*n* = 63) of the isolates were resistant to both imipenem and meropenem, and 64% (*n* = 74) were resistant to colistin (Table S5). Of these, 63 strains carried two *bla*_NDM_ genes (*bla*_NDM-5_ [*n* = 62] and *bla*_NDM-1_ [*n* = 1]), whereas 44 strains harbored four *mcr* genes (*mcr-1* [*n* = 26], *mcr-3* [*n* = 8], *mcr-8.2* [*n* = 7], and *mcr-9* [*n* = 3]). Coexistence of *bla*_NDM-5_ and *mcr* genes was identified in 28 isolates (15 *K*. *pneumoniae*, 10 *E. coli,* and 3 *E. cloacae*), including *bla*_NDM-5_/*mcr-1* (6 *K. pneumoniae and 9 E. coli*), *bla*_NDM-5_/*mcr-3* (5 *K. pneumoniae and 1 E. coli*), *bla*_NDM-5_/*mcr-8* (4 *K*. *pneumoniae*), and *bla*_NDM-5_/*mcr-9* (3 *E. cloacae*) (Table S1).

MLST analysis revealed that 69 *K*. *pneumoniae* isolates belonged to 17 distinct sequence types, with the most common being ST152 (*n* = 10), ST395 (*n* = 9), and ST709 (*n* = 6). The remaining 12 *K*. *pneumoniae* isolates could not be assigned to any known sequence type. Among the 37 *E. coli* isolates, 13 distinct sequence types were identified, including ST226 (*n* = 8), ST1286 (*n* = 7), and ST11738 (*n* = 6). The eight *Enterobacter cloacae* isolates were assigned to ST1 (*n* = 7) and ST932 (*n* = 1), whereas the two *Citrobacter freundii* isolates were classified as ST332 (*n* = 2) (Table S1).

### Extensive distribution of diverse ARGs and plasmids in Chinese chicken farms

Our analysis identified a total of 132 unique ARGs across all strains, with 2,452 total detections across all isolates. Nearly all isolates (118/119) exhibited an MDR profile, each harboring at least 10 ARGs (Table S1). The most frequently detected ARGs included *floR*, *sul2*, *sul1*, *tet*(A), *aph*(3')*-Ia*, *dfrA12*, *mph*(A), *aadA2*, *aph* (6)*-Id*, and *bla*_NDM-5_ (Table S1). These ARGs are associated with the antibiotics frequently used in chicken farms, such as florfenicol (*floR*), sulfonamide/trimethoprim (*sul1*, *sul2*, and *dfrA12*), tetracyclines (*tet*(A)), aminoglycosides (*aph*(3')*-Ia*, *aadA2*, and *aph (6)-Id*), and macrolides (*mph*(A)).

We identified 40 distinct types of beta-lactam resistance genes, including *bla*_CMH_, *bla*_CMY,_*bla*_CTX-M_, *bla*_DHA_, *bla*_EC_, *bla*_LAP_, *bla*_OXA_, *bla*_SHV_, and *bla*_TEM_. Among the detected β-lactam resistance genes, *bla*_CTX-M_ (76.4%) and *bla*_TEM_ (58.0%) were the most prevalent β-lactam resistance genes, and *bla*_SHV_ had the highest diversity in its nucleotide sequence, with seven types of sequences present in the isolates. Almost all sequenced isolates harbored more than two plasmids (117/119). A total of 132 unique plasmid replicon types were detected among all of the strains, including Inc. (FIA, FIB, FII, R, X3) and Col (RNAI, MG828, 440I, 156). The most frequently detected plasmids among all isolates included IncF (*n* = 254), followed by Col (*n* = 188) and IncX3 (*n* = 57) (Table S2). The putative plasmid-borne ARGs predicted by PlasFlow were presented in Table S3. Overall, we identified a high diversity of plasmid-borne ARGs (1,633 cumulative hits in total), which accounted for 66.6% (1,633/2,452) of the number of total detected ARGs. Obviously, the transmission of ARGs among different microorganisms in chicken farms was mainly driven by a variety of plasmids, which facilitate the horizontal transfer of ARGs. The most prevalent plasmid-borne ARG was the florfenicol resistance gene *floR*, followed by *tet(A*), *sul1*, *aph(3')-Ia*, *bla*_TEM-1_, and *sul2* (Table S3).

### Characterization of NDM-5 carrying IncFII plasmid

**Fig 2:**
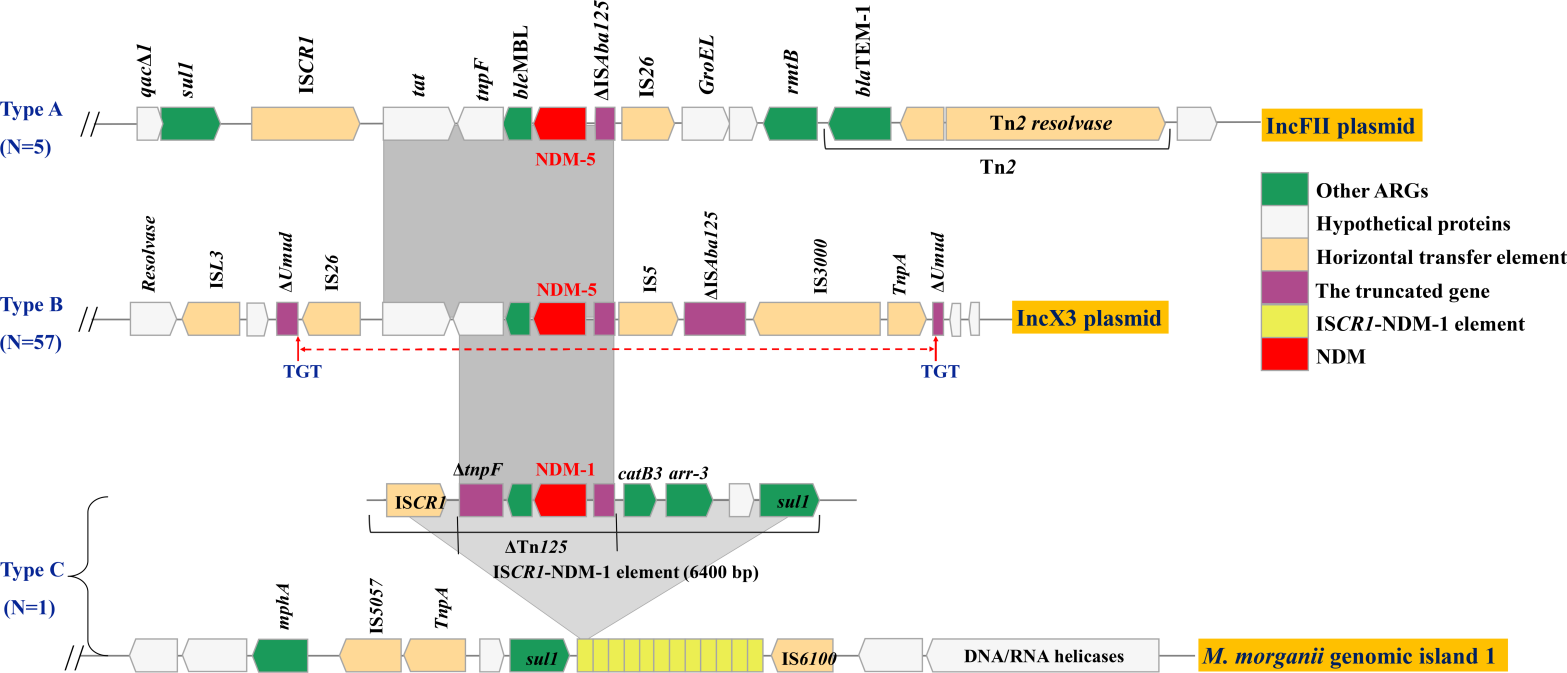
Comparison of the *bla*_NDM_-containing regions from different plasmids and the chromosome. Genes and ORFs are shown as arrows, and their orientations of transcription are indicated by the arrowheads. Hypothetical protein is indicated by white arrows, horizontal transfer element is indicated by orange arrows, IS*CR1*-NDM-1 element is indicated by yellow arrows, *bla*_NDM_ is indicated by red arrows, and other ARGs are indicated by green arrows. The truncated genes are indicated by purple bars. Shared regions with above 99% identity are indicated by light-gray shading.

The 63 NDM-positive contigs obtained via WGS were categorized into three types (A–C) based on the analysis of the genetic structures immediately surrounding the *bla*_NDM_ gene and the associated plasmid replicon types (or chromosomal location). Type B, associated with the IncX3 replicon and a characteristic flanking context, was the most common (*n* = 57). Type A represented contexts found on IncFII plasmids (Type A), whereas Type C corresponded to the chromosome-borne *bla*_NDM-1_ in *Morganella morganii* (Type C). We obtained the complete IncFII plasmid of strain GDEC-PN45 using Illumina and Nanopore sequencing. This plasmid was 96,419 bp in length, with 101 predicted coding sequences (CDSs). Online BLAST against the NCBI nr database revealed that it shared an overall similar backbone with plasmid p_dm884b_NDM5 (CP096181), p_b148b_NDM5 (CP095579), p1ESCUMpO83_CORR (CP034254), pNDM-SDCRK18-7 (MN641485), and pSDCDK-IncFNDM5 (MT621569). A large ~15 kb MDR region was detected in our plasmid. Abundant AMR genes and mobile elements, including *bla*_NDM-5_, *rmtB*, *bla*_TEM-1B_, *aadA2*, *dfrA12*, *sul1,* IS*26,* IS*CR1,* IS*Pa40,* Tn*3,* and Tn*As3,* were concentrated in this region (Fig. S1A). The *bla*_NDM-5_ gene was located in a structure of *qac*Δ*1-sul1*-IS*CR1-tat-tnpF-ble*_MBL_-*bla*_NDM-5_-ΔIS*Aba125*-IS*26-GroEL-rmtB-bla*_TEM-1_, which shared 100% nucleotide identity with the corresponding region of plasmid p_dm884b_NDM5 (CP096181). Comparative genomic analysis showed that it shared an identical *bla*_NDM-5_ carrying region, *tat-tnpF-ble*_MBL_-*bla*_NDM-5_-ΔIS*Aba125,* with our IncX3 plasmid (Fig. 2).

### Widespread *bla*_NDM-5_ gene via an IncX3-type plasmid

The IncX3 *bla*_NDM-5_-carrying plasmids were present in 57 strains, including 27 *K. pneumoniae*, 20 *E. coli*, 8 *E. cloacae,* and 2 *C. freundii*. The *bla*_NDM-5_-carrying IncX3 plasmid in strain GDKL-149-1 was completely sequenced; it had a length of 46,161 bp, had an average GC content of 46.6%, and showed 100% nt identities to *E. coli* strain EC7 plasmid pEC7-NDM-5 (CP060966) (Fig. S1B). The backbone of the other 56 plasmids was identical to the complete sequence of the IncX3 plasmid in strain GDKL-149-1, indicating that these IncX3 plasmids have highly conserved plasmid backbones. The *bla*_NDM-5_ gene was in the typical genetic context IS*26-tat-tnpF-ble*_MBL_-*bla*_NDM-5_-ΔIS*Aba125*-IS*5-*ΔIS*Aba125*-IS*3000-TnpA* found in IncX3-type plasmids, and no other ARGs were detected in these plasmids. Of note, the *bla*_NDM-5_ harboring region bracketed by IS*26* and *TnpA* was inserted into the *umuD* gene, leading to the flanking 3 bp direct repeats (TGT) (Fig. 2). The frequent discovery of these *bla*_NDM-5_-bearing IncX3 plasmids in China demonstrated that IncX3 plasmids were the predominant epidemic vehicles mediating dissemination of the *bla*_NDM–5_ in China (24).

The chromosome-borne *bla*_NDM-1_ was detected in a highly drug-resistant *M. morganii* strain GDMM86. A novel Tn*7*-like transposon (Tn*6835*) and a peculiar genomic island (MMGI-1) were identified in GDMM86, and to our surprise, we discovered that *bla*_NDM-1_ exists on MMGI-1 as multiple copies with IS*CR1* in the form of IS*CR1-bla*_NDM-1_ tandem repeats (25).

### Diverse genetic contexts and plasmid vehicles of *mcr-1* genes

The genetic context of *mcr* is heterogeneous and diverse. Considering the impact of plasmid types on the replication and horizontal transfer efficiency of the *mcr* gene, as well as the varying degrees of risk associated with *mcr*-flanking sequences during co-transfer, we classified these *mcr*-carrying contigs based on their plasmid types and *mcr*-flanking sequences. Specifically, 44 *mcr*-carrying contigs obtained via WGS showed eight genomic backbone profiles: type D (*mcr-1*, *n* = 6, two species, IncI2 plasmid), type E (*mcr-1*, *n* = 1, *E. cloacae*, IncX4 plasmid), type F (*mcr-1*, *n* = 7, *K. pneumoniae*, IncHI2 plasmid), type G (*mcr-1*, *n* = 3, *K. pneumoniae*, IncHI2 plasmid), type H (*mcr-1*, *n* = 9, *K. pneumoniae*, IncY plasmid), type I (*mcr-3*, *n* = 8, *K. pneumoniae* and *E. coli*, novel plasmid), type J (*mcr-8*, *n* = 7, *K. pneumoniae,* IncFII/IncR plasmid), and type K (*mcr-9*, *n* = 3, *E. cloacae*, IncHI2 plasmid).

The IncI2-type *mcr-1*-carrying plasmids were present in 6 *E. coli* strains (GDEC-N23, GDEC-N 44, GDEC-P95-3, GDEC-N17, GDEC-PN45, and GDEC-799). We obtained the complete IncI2 plasmid of strain GDEC-PN45 by Illumina and Nanopore sequencing. This plasmid was 62,594 bp in size, comprising 81 coding sequences (CDSs), and shared high sequence identity with *mcr-1*-positive plasmid pAH01-2 (93% query coverage and 99% identity) (CP055253), which was isolated from a human in China (Fig. S2A). The *mcr-1*-carrying IncI2 plasmid lacks the IS*Apl1* element in the vicinity of the *mcr-1* gene. However, the *mcr-1–pap2* element was identified between the *top* (encoding DNA topoisomerase III) and *nikB* (relaxase) genes (Fig. 2).

The *mcr-1*-harboring IncX4 plasmid was present in *E. coli* strain GDEC-36. Bioinformatics analysis identified that *mcr-1* was located in a 30,218 bp length contig, with a GC content of 41.8% (Fig. S2C). Genome alignments indicated that this contig was identical to the plasmid pMCR-NMG38 (100% query coverage and 100% identity query) (MK836307), isolated from an *E. coli* strain in China, and nearly identical to plasmid PNUSAS037276_2 (99% query coverage and 99.95% identity) (CP092309), isolated from a *Salmonella enterica* strain in the United States. The *mcr-1* gene is part of an approximately 2.6 kb *mcr-1-pap2* element. Except for the *mcr-1* colistin resistance gene, no other resistance genes were detectable; in addition, the insertion element IS*Apl1* flanking the *mcr-1* gene was not detected.

Ten *K. pneumoniae* strains harbored an IncHI2 plasmid contig, seven of which (GDKL-69-1, GDKL-76, GDKL-57, GDKL-34-1, GDKL-25, GDEC-38, and GDKL-Q8) had the same contig size of ~96 kb (Type F), and the remaining strains (GDKL-2, GDKL-26-1, and GDKL-Q5) had a contig size of ~198 kb (Type G). Strain GDKL-34-1 harbored a *mcr-1*-carrying contig of size 97,493 bp (Fig. S2B). BLASTn analysis showed that this contig has a query coverage of 95% and maximal 99% identity to *E. coli* strain AMSCJX04 plasmid pAMPD03 (CP058311). The *mcr-1* gene was located on the contig within the genetic context IS*Apl1-mcr-1*-Δ*pap2* (211 bp remnant). In addition to *mcr-1*, *bla*_CTX-M-64_ and IS*Ecp1* also localize to this contig, and co-localization of *mcr-1* with *bla*_CTX-M-64_ on a single plasmid contig could accelerate the dissemination of both genes through co-selection. Strain GDKL-2 had the largest size of the InHI2 plasmid contig with a length of 198,587 bp and predicted CDSs of 225 (Fig. S2D). The BLASTn program revealed that this contig has 99% nucleotide identity and 100% query coverage with the sequence of the *Salmonella* SH16G1369 strain plasmid pSH16G1369 (accession no MH522418). It is speculated that *mcr-1* is initially captured and mobilized by the Tn*6330*, and then, IS*Apl1* begins to be lost over time, leading to the formation of a diverse genetic structure of *mcr-1* (26). Sequence analysis revealed that *mcr-1* was located between an IS*Apl1* and a complete *pap2* encoding gene, forming an “IS*Apl1-mcr-1-pap2*” element.

**Fig 3:**
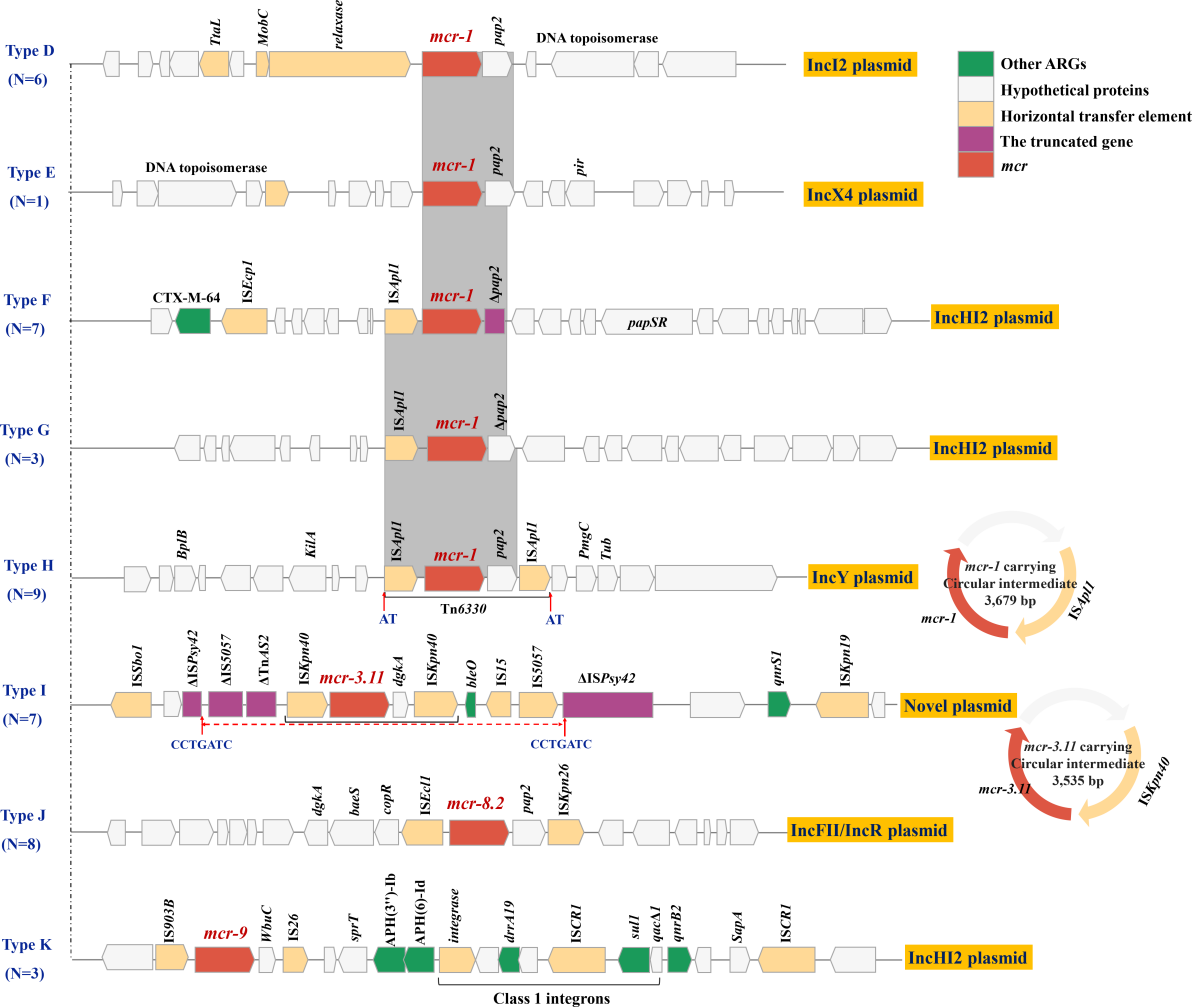
Schematic representation of the *mcr*-containing regions from different plasmids. Genes and ORFs are shown as arrows, and their orientations of transcription are indicated by the arrowheads. Hypothetical protein is indicated by white arrows; horizontal transfer element is indicated by orange arrows, *mcr* is indicated by pink arrows, and other ARGs are indicated by green arrows. The truncated genes are indicated by purple bars. Shared regions with above 99% identity are indicated by light-gray shading.

Nine strains (eight *K*. *pneumoniae* and one *E. coli*) harbored a phage-like IncY plasmid contig. The complete plasmid sequence of GDKL-684 was found to be 96,471  bp in length and exhibit a GC content of 47.3% (Fig. S3A). It was assigned to the IncY group and encodes a total of 97 open reading frames. BLASTn results showed that it was 98% identical to phage P1 (AF234172) at 71% coverage and phage P7 (NC_050152) at 73% coverage. It exhibited high-level homology to the *mcr-1*-carrying phage-like plasmid pHYEC7-mcr1 (KX518745) in *E. coli*, with 98.9% identity at 90% coverage. The complete sequence of plasmid GDKL-684 indicates that *mcr-1* is located on the transposon Tn*6330* (IS*Apl1-mcr-1-orf*-IS*Apl1*), which is inserted into a genetically conserved region of the plasmid backbone, resulting in a pair of 2 bp direct repeat sequence (AT). Previous studies have shown that Tn*6330* can mediate *mcr-1* translocation into various plasmid backbones by forming a circular intermediate (3,679 bp) (27). In this study, we also verified the existence of this *mcr-1*-carrying circular intermediate by nested PCR and Sanger sequencing (Fig. 3) (27).

### Characterization of an *mcr-3*-carrying plasmid lacking a known replicon type

Seven *K. pneumoniae* and one *E. coli* strain harbored an *mcr-3.11*-carrying plasmid. To characterize the genetic environment of *mcr-3.11*, we performed Nanopore and Illumina hybrid sequencing of strain GDKL-149-1 and obtained a 76,065 bp plasmid with an average GC content of 52.7%, containing 92 predicted open reading frames (ORFs), including two additional resistance genes (*qnrS1* and *bleO*) (Fig. S3B). The Blastn program showed that this plasmid has 92% nucleotide identity and 100% query coverage with the sequence of *K. pneumoniae* strain MYKLB95 plasmid MYKLB95-1 (Accession No. MH341574). Notably, *mcr-3.11* was located within a composite transposon structure with the gene arrangement IS*Kpn40-mcr-3.11-dgkA*-IS*Kpn40* and a potentially mobile 3,535 bp circular intermediate consisting of the *mcr-3.11,* and an IS*Kpn40* element was validated by inverse PCR as previously described (28). The *mcr-3.11*-carrying region bracketed by IS*5057* and ΔIS*5057* was inserted into the IS*Psy42*, resulting in a pair of 7 bp direct repeats (CCTGATC) (Type I). This *mcr-3.11*-carrying plasmid lacks any known replicons identifiable by the PlasmidFinder database and is presumed non-conjugative due to the absence of a complete set of *tra*-related proteins (Fig. 3).

### *mcr-8* was located on a large IncFII/IncR hybrid plasmid

Eight *K. pneumoniae* strains harbored an *mcr-8.2*-carrying plasmid, and the strain GDKL-555 was subjected to Nanopore and Illuminate hybrid sequencing. In strain GDKL-555, *mcr-8.2* is located on an 82,768 bp, IncFII/IncR hybrid plasmid encoding 98 predicted ORFs with a GC content of 52.5% (Fig. S3C). It was 99.92% identical to *K. pneumoniae* strain KP700 plasmid pKP700 (OL804393) at 97% coverage. Further analysis found that *mcr-8* co-localizes with other antimicrobial resistance genes *bla*_OXA-1_, *emrE*, *aacA4*, *catA2*, and *floP* on the same plasmid, thereby allowing possible co-selection. Previous studies have indicated that IS*903B* and IS*Ecl1* play critical roles in the horizontal transmission of *mcr-8* among Enterobacterales (29, 30). Linear alignment of the *mcr-8.2-*carrying regions indicated that *mcr-8.2* in our study was within the genetic structure *dgkA-baeS-copR*-IS*Ecl1-mcr-8.2-pap2*-IS*Kpn26* (31), which was different from *dgkA-baeS-copR-mcr-8.2-pap2* in plasmid pKP91 (Fig. 3).

### The spread of *mcr-9* is driven by the IncHI2 “superplasmids.”

Three *E. cloacae* strains harbored an *mcr-9*-carrying plasmid. To fully understand the genetic profile of *mcr-9*, we extracted total DNA from *E. cloacae* GDEC-490 and sequenced it using Nanopore and Illumina platforms. Finally, we obtained *mcr-9*-carrying circular InHI2 “superplasmids” of size 381,054 bp with a GC content of 48.7%; in addition to *mcr-9*, it also contained other antibiotic resistance genes, including *bla*_OXA-10_, *aadA1*, *aac(6")-lb-cr*, *aph(3')-Ia*, *ant(2'')-Ia*, *sul1*, *mph(A*), *catB8,* and *dfrA12* (Fig. S3D). The close association between *mcr-9* and InHI2 plasmids was confirmed in the initial report of *mcr-9* (32, 33) and in multiple subsequent studies on *mcr-9* (34–37). The *mcr-9* was inserted into the genetic structure composed of *rcnR-rcnA-pcoE-pcoS*-IS*903B*-like-*mcr-9-wbuC*-IS*26*, and other mobile genetic elements such as IS*Bcen27*, IS*Kpn8*, IS*103*, IS*1R*, IS*5D*, IS*Stma11*, IS*5057*, IS*4321R*, IS*26*, and Tn*As3* were also found (Fig. 3), which may indicate the recombination activity of this plasmid.

## DISCUSSION

Antimicrobial resistance among gram-negative bacteria is a significant global health concern, particularly with the increase in multidrug-resistant strains in food-producing animals like poultry. The extensive use—and often misuse—of β-lactams and colistin in Chinese poultry production may be a primary driver of this worrying phenomenon. Understanding the genetic diversity and mechanisms behind the dissemination of resistance genes in these bacteria is crucial for developing effective control strategies.

In this study, we demonstrated the extensive genetic diversity of CR and col-R gram-negative strains isolated from Chinese commercial chicken farms. Whole-genome sequencing of 119 gram-negative isolates revealed 17 sequence types (STs) of *K. pneumoniae* and 13 STs of *E. coli*, indicating a high level of genetic variability. Nearly all isolates (118/119) exhibited multidrug resistance profiles, each harboring at least 10 ARGs.

A recent investigation reported a significant prevalence of the *bla*_NDM_ gene among Enterobacteriaceae isolates from broiler chickens, with detection rates between 52.9% and 72.9% (38). In contrast, Liu et al. observed an even higher antimicrobial resistance rate of 78.6% in poultry flocks (39). Notably, all isolates carried at least two β-lactamase genes, including *bla*_CMH_, *bla*_CMY,_*bla*_CTX-M_, *bla*_DHA_, *bla*_EC_, *bla*_LAP_, *bla*_OXA_, *bla*_SHV_, and *bla*_TEM_. Consistent with previous reports (40–42), the *bla*_CTX-M_ type was the dominant β-lactam resistance gene, present in 76.4% of the isolates. Specifically, CTX-M-55 was the most prevalent genotype (36.1%), followed by *bla*_CTX-M-65_ (14.3%) and *bla*_CTX-M-27_ (11.8%). These findings align with recent surveys showing a rapid increase in β-lactamase-producing *E. coli* from poultry sources in China (41, 43).

Despite carbapenems not being authorized for use in chicken farms, we detected a widespread *bla*_NDM-5_ among the isolates (44, 45). This suggests that the use of β-lactam antibiotics, which are structurally similar to carbapenems, may exert indirect selective pressure for the accumulation of *bla*_NDM_ genes. Our results identified IncX3 plasmids as the major vectors for *bla*_NDM_, corroborating previous studies that highlight the clinical importance of these plasmids in facilitating horizontal gene transfer of various *bla*_NDM_ variants (46). Alarmingly, there are increasing reports of the identification of IncX3-*bla*_NDM-5_ plasmid in human commensal and pathogenic strains (47, 48). The IncX3 plasmid is highly conjugated and stable (49, 50) and does not incur adaptability costs to its bacterial host, making it a significant vector for *bla*_NDM_ genes. The widespread prevalence of IncX3-*bla*_NDM-5_-carrying bacteria in chicken farm environments may be linked to their increasing colonization in humans and warrants further investigation.

Beyond its prevalence, the highly conserved genetic context of blaNDM-5 observed on the predominant IncX3 plasmids warrants discussion. The typical IS*26-tat-tnpF-ble*_MBL_-*bla*_NDM-5_-ΔIS*Aba125*-IS*5-*ΔIS*Aba125*-IS*3000-TnpA* structure, often inserted within the *umuD* gene via transposition as evidenced by target site duplications (Fig. 2), underscores a highly successful mobilization unit that has likely undergone extensive horizontal transfer and clonal expansion within this environment. The stability and high conjugative efficiency often associated with IncX3 plasmids further explain their role as potent vectors for *bla*_NDM-5_ (48). Although less common, the detection of *bla*_NDM-5_ on IncFII plasmids, sharing core resistance module components, suggests potential inter-plasmid transfer of the resistance determinant. This contrasts sharply with the unique chromosomal integration of *bla*_NDM-1_ via an IS*CR1*-mediated mechanism within a genomic island in *M. morganii*, highlighting diverse evolutionary pathways for NDM gene dissemination.

Historically, colistin has been widely used in animal production as both a curative treatment and a growth promoter (51). Although its use as a growth promoter has been banned in China since 2017 (52), the extensive prior use of colistin not only altered the composition of bacterial populations but also contributed to the persistence of *mcr-1* in farm environments (53). Previous studies have shown that the abundance of *mcr-1* in chicken farms did not significantly decrease following the ban (53). In our study, whole-genome sequencing revealed the occurrence of *mcr-1*, *mcr-3*, *mcr-8,* and *mcr-9* in various chicken-derived gram-negative isolates. We characterized up to eight genetic contexts of *mcr* genes in multiple incompatibility (Inc) groups of plasmids. The genetic contexts showed significant diversity, involving various insertion sequences, transposable elements, plasmid replication proteins, and other resistance determinants. This high flexibility indicates that commercial chicken farms remain a hotspot for active horizontal transfer of *mcr* genes.

In stark contrast to the relatively uniform *bla*_NDM_ context, the genetic environments surrounding the detected mcr genes (*mcr-1*, *mcr-3*, *mcr-8*, and *mcr-9*) exhibited remarkable diversity (Types D–K). This heterogeneity, involving a wide array of plasmid backbones (IncI2, IncX4, IncHI2, IncY, IncFII/IncR, and a plasmid lacking a known replicon type), strongly suggests multiple independent acquisition and mobilization events occurring within the poultry farm setting. The varied structural arrangements around mcr-1, particularly the presence, absence, or truncation of IS*Apl1* (Fig. 3), align with the proposed model of Tn*6330*-mediated transposition followed by element decay (26), a mechanism further supported by our confirmation of the Tn*6330* circular intermediate. Similarly, the identification of IS*Kpn40* flanking *mcr-3.11* and its circular intermediate, and the role of IS*Ecl1* near *mcr-8.2*, highlight the critical involvement of diverse insertion sequences in mobilizing different *mcr* variants. Furthermore, the co-localization of *mcr-1* with *bla*_CTX-M-64_ (IncHI2) and *mcr-8.2* or *mcr-9* with numerous other ARGs on complex plasmids underscores the potential for co-selection, which could maintain these critical resistance genes even under reduced colistin usage pressure. These findings collectively portray poultry farms as dynamic platforms for the evolution and dissemination of *mcr* genes via diverse plasmid vehicles and mobile elements.

Significantly, *bla*_NDM-5_ co-existed with different *mcr* genes in several isolates: *mcr-1* (nine *E. coli* and six *K*. *pneumoniae*), *mcr-3* (five *K*. *pneumoniae* and one *E. coli*), *mcr-8* (four *K*. *pneumoniae*), and *mcr-9* (three *E. cloacae*). Overall, the combination of *bla*_NDM-5_ and *mcr-1* was the most dominant (53.6%, 15/28) among NDM-MCR strains. To our knowledge, this is the first report of chicken-origin *E. cloacae* isolates from China co-harboring *mcr-9* and *bla*_NDM–5_. Although previous studies have sporadically reported the co-existence of *bla*_NDM-5_ with *mcr-1* (54–56), *mcr-3* (57), *mcr-8* (58), and *mcr-9* (59), the occurrence of these genes in single bacterial strains is still relatively rare worldwide. These “superbugs,” capable of co-producing MCR and NDM-5, were non-susceptible to most or even all antimicrobial drugs tested. It is particularly concerning that these isolates may spread further through the animal–human chain into medical facilities and among high-risk patients, potentially leading to untreatable infections.

### Conclusion

This study provides comprehensive evidence of the co-occurrence of *bla*_NDM-5_ and multiple *mc*r genes in gram-negative bacteria from poultry farms in China. Our findings highlight the critical role these farms play as reservoirs for multidrug-resistant bacteria, which can potentially transfer to humans and cause untreatable infections. Immediate action is required to implement stricter antibiotic usage policies in poultry production and to enhance surveillance programs. Such measures are essential to curb the dissemination of these superbugs and protect public health. The persistence and potential spread of these gram-negative bacteria in chicken farm environments pose significant health risks that deserve immediate attention. Our results contribute to a better understanding of antimicrobial resistance genes on chicken farms and may assist governmental agencies in formulating strategies to mitigate this public health threat.

## Data Availability

All WGS data obtained in this study have been deposited in the NCBI BioProject database: PRJNA836393 and PRJNA663257.

## SUPPLEMENTAL MATERIAL

Figure S1 spectrum.02900-24-s0001.pdf Schematic diagram of two plasmids carrying *blaNDM-5*.

Supplemental tables spectrum.02900-24-s0002.xlsx Tables S1 to S5.

ASM does not own the copyrights to Supplemental Material that may be linked to, or accessed through, an article. The authors have granted ASM a non-exclusive, world-wide license to publish the Supplemental Material files. Please contact the corresponding author directly for reuse.
