# Supplementary material for: Whole genome analysis reveals the distribution and diversity of plasmid reservoirs of NDM and MCR in commercial chicken farms in China
Source: Microbiol Spectr. 2025 Jun 9;13(7):e02900-24. doi: 10.1128/spectrum.02900-24 (PMC12210879; doi:10.1128/spectrum.02900-24)
Supplement: Figure S1 — Schematic diagram of two plasmids carrying blaNDM-5. [file spectrum.02900-24-s0001.pdf]

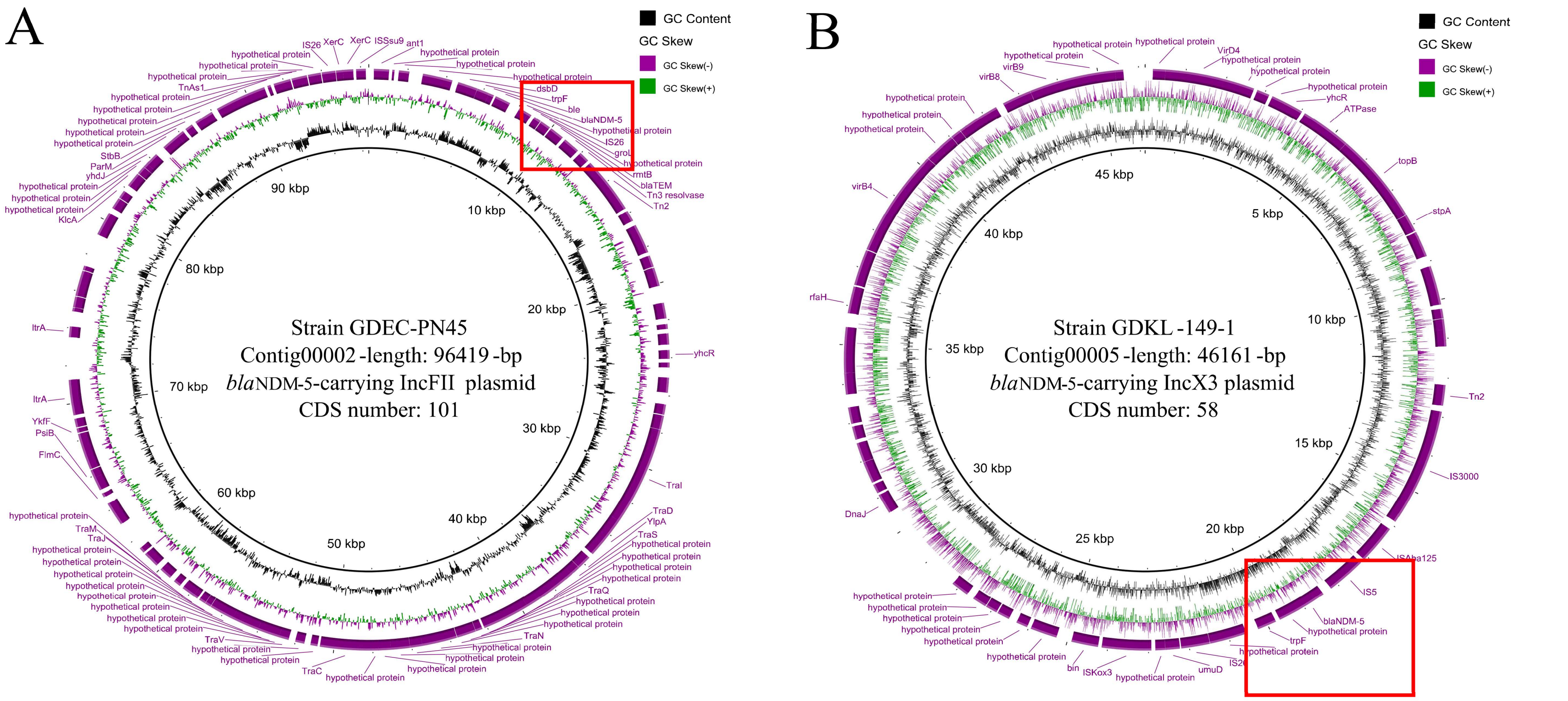

Figure S1. Schematic diagram of two plasmids carrying *bla*NDM-5. The colored arrows in the outer ring depict various gene families. The circles display (from outer to inner) the predicted coding sequences, GC content, GC skew, and scale (in kilobases).

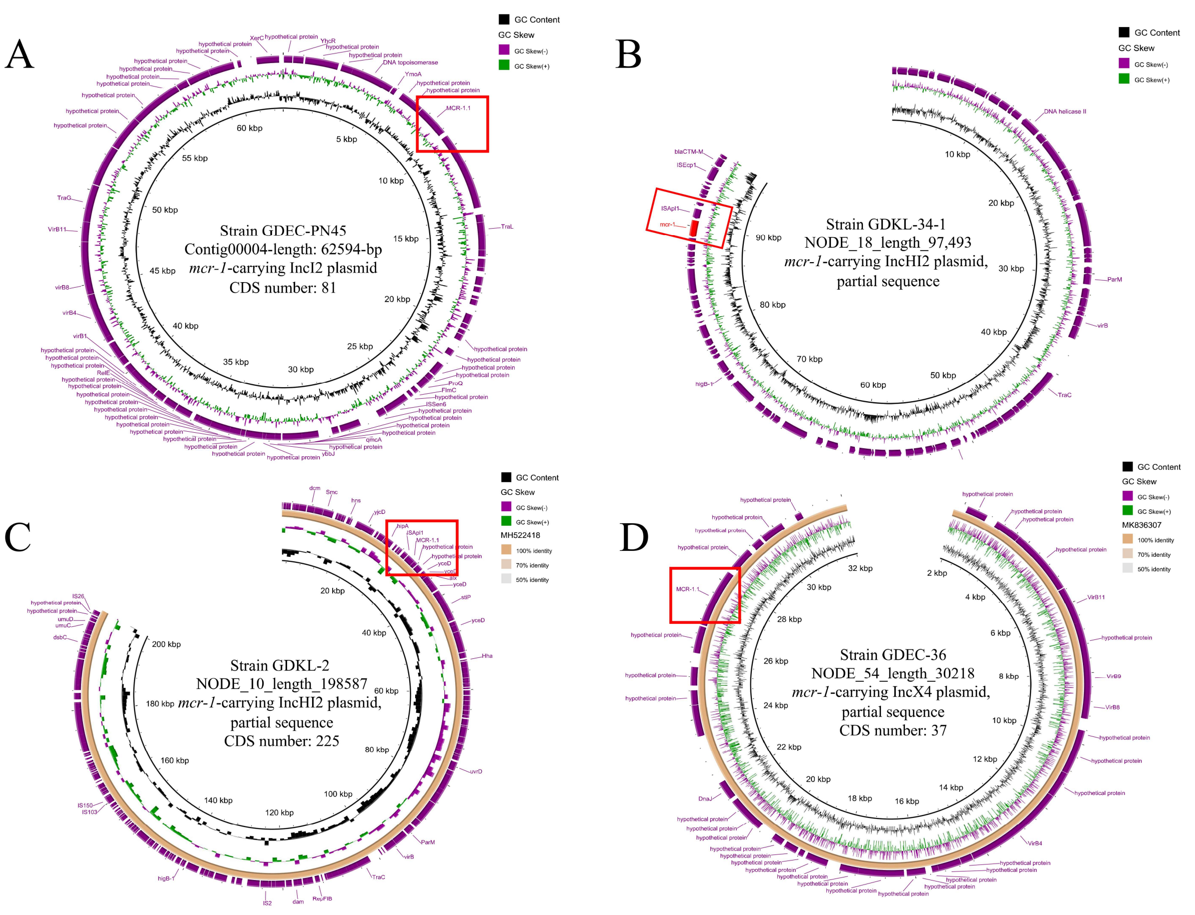

Figure S2. Schematic diagram of four plasmids carrying *mcr-1*. The colored arrows in the outer ring depict various gene families. The circles display (from outer to inner) the predicted coding sequences, GC content, GC skew, and scale (in kilobases).

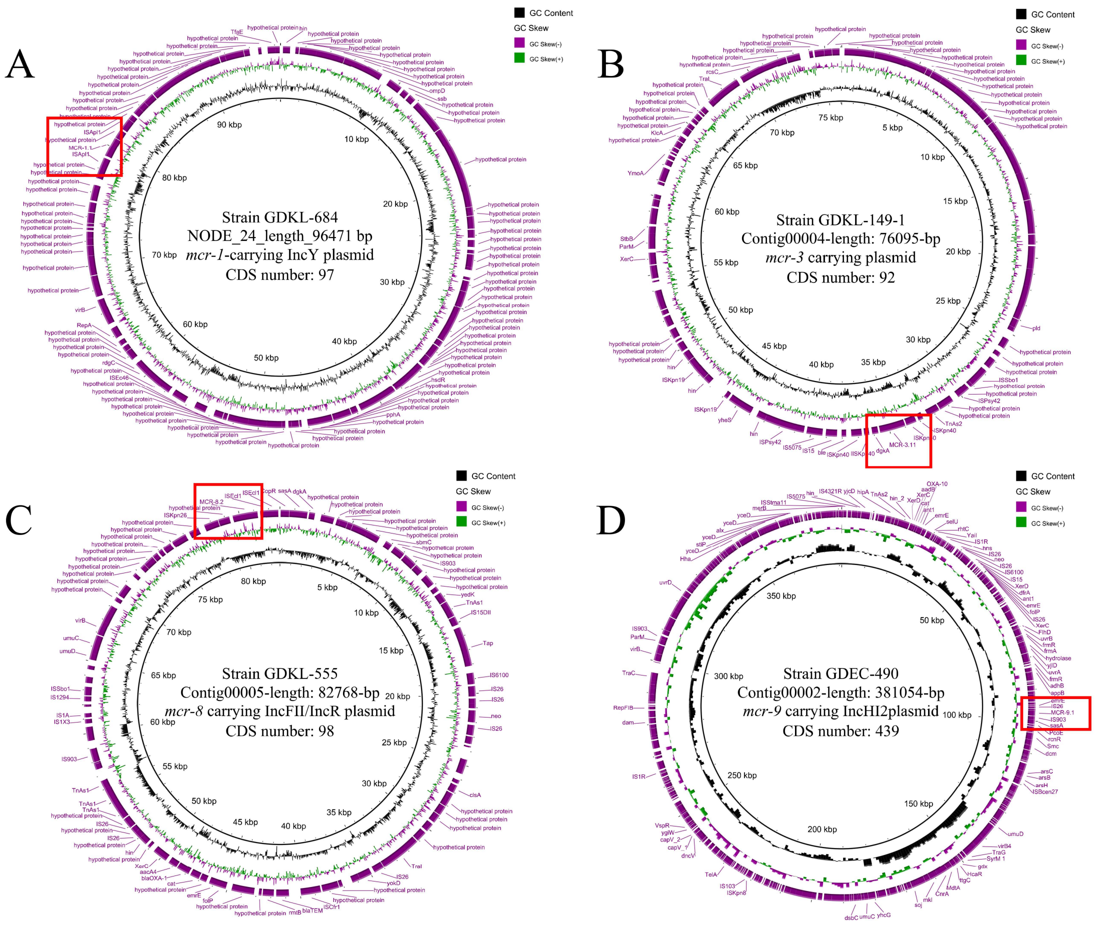

Figure S3. Schematic diagram of four plasmids carrying *mcr-1*, *mcr-3*, *mcr-8* and *mcr-9*. The colored arrows in the outer ring depict various gene families. The circles display (from outer to inner) the predicted coding sequences, GC content, GC skew, and scale (in kilobases).
